# A comprehensive evaluation of the health benefits of forest therapy for cancer survivors

**DOI:** 10.1265/ehpm.25-00333

**Published:** 2026-01-28

**Authors:** Renn-Shiuan Wei, Yi-Shun Chu, Ming-Yu Hong, Ming-Yi Li, Su-Wei Fan, Gene-Sheng Tung, Chung-Hua Hsu

**Affiliations:** 1 Department of Chinese Medicine, Branch of Linsen, Chinese Medicine, and Kunming, Taipei City Hospital, Taipei, Taiwan; 2 Institute of Traditional Medicine, College of Medicine, National Yang Ming Chiao Tung University, Taipei, Taiwan; 3 Institute of Health and Welfare Policy, National Yang Ming Chiao Tung University, Taipei, Taiwan; 4 Fushan Research Center, Taiwan Forestry Research Institute, Ministry of Agriculture, Taiwan; 5 Forest Ecology Division, Taiwan Forestry Research Institute, Ministry of Agriculture, Taiwan; 6 Department of Traditional Chinese Medicine, National Yang Ming Chiao Tung University Hospital, Yilan, Taiwan; 7 School of Chinese Medicine, College of Medicine, National Yang Ming Chiao Tung University, Taipei, Taiwan

**Keywords:** Forest therapy, Forest bathing, Cancer survivors, Physiological indicators, Mood disturbance, Stress reduction

## Abstract

**Background:**

While advancements in cancer treatment have improved survival rates, some survivors continue to experience emotional difficulties that adversely affect overall well-being. Forest therapy, a nature-based intervention, has demonstrated potential in alleviating psychological and physiological stress.

**Methods:**

This single-arm pre–post study evaluated a two-hour guided forest therapy session in a submontane forest in Taiwan. Participants were recruited via posters and online registration forms disseminated by the Linsen Chinese Medicine and Kunming Branch (LCMKB) of Taipei City Hospital and were screened for eligibility according to predefined inclusion and exclusion criteria. Outcomes included psychological measures—the *Profile of Mood States 2nd Edition–Adult Short (POMS 2-A)*, *Beck Depression Inventory–Second Edition (BDI-II)*, and *Beck Anxiety Inventory (BAI)*—along with physiological indicators (heart rate, blood pressure, heart rate variability, salivary cortisol).

**Results:**

Of the 40 participants, 38 completed the program. Significant improvements were observed in mood disturbance (*The Total Mood Disturbance of POMS 2-A*: 4.0 → −8.0), anxiety (*BAI*: 5.0 → 3.5), depression (*BDI-II*: 5.5 → 4.0), and salivary cortisol levels (1.07 → 0.42 µg/dL), all with large effect sizes. Regression analyses suggested that baseline emotional status and certain demographic factors may have influenced outcomes. Participants with higher initial anxiety and depression, younger age, and shorter cancer duration appeared to show relatively greater improvements.

**Conclusions:**

This first study of forest therapy among cancer survivors in Taiwan provides preliminary evidence that guided forest therapy may be a useful complementary approach for enhancing emotional well-being and reducing physiological stress. Significant improvements were observed across multiple psychological measures (*POMS 2-A*, *BAI*, *BDI-II*) and in salivary cortisol. Benefits were more pronounced among participants with higher baseline distress, younger age, or shorter cancer duration, underscoring the importance of considering baseline health profiles and demographic characteristics when designing interventions.

**Trial Registration:**

Approved by the Taipei City Hospital Research Ethics Committee (TCHIRB-11104010). ClinicalTrials.gov Identifier: NCT06001723.

**Supplementary information:**

The online version contains supplementary material available at https://doi.org/10.1265/ehpm.25-00333.

## 1. Introduction

According to the World Health Organization’s (WHO) International Agency for Research on Cancer (IARC) 2022 global cancer data, statistics on the latest incidence, mortality, and trends of 36 types of cancer across 185 countries were presented. The report indicated that in 2022, approximately 20 million new cancer cases were diagnosed worldwide, with nearly 9.7 million cancer-related deaths. Approximately 20% of people will be diagnosed with cancer during their lifetime, and around 1 in 9 men and 1 in 12 women will succumb to the disease [1]. In Taiwan, according to the Ministry of Health and Welfare’s 2022 statistics, the top three causes of death were cancer, heart disease, and Coronavirus disease 2019 (COVID-19). Cancer remains the leading cause of death, accounting for 24.9% of total deaths. Among male cancer patients, lung cancer ranks first, followed by liver cancer and colorectal cancer; among female cancer patients, lung cancer is also the most common, followed by colorectal cancer and breast cancer [2].

The increasing incidence of cancer, along with improved post-treatment survival rates, has gradually transformed cancer into a “chronic disease.” [3] However, large-scale studies show that cancer patients generally experience higher levels of negative emotions compared to the general population [4]. The American Society of Clinical Oncology (ASCO) recently updated its guidelines for managing anxiety and depression in adult cancer survivors, emphasizing that stress is closely associated with depressive and anxiety symptoms, physical complaints, treatment-related morbidities, and even increased cancer mortality [5]. Thus, alleviating negative emotions in post-surgery cancer patients is crucial. Moreover, the risk of cancer recurrence itself can be a major source of psychological distress, particularly in cancers with high five-year survival rates but persistent relapse potential [6]. Depression is particularly common among breast cancer patients, with epidemiological data showing that up to 30% experience depressive symptoms [7], and survivors facing a 39% higher risk of depression than non-cancer controls [8]. The first year after diagnosis carries the greatest risk, with adjuvant treatments such as chemotherapy and endocrine therapy identified as key contributing factors [8]. Moreover, stress can exacerbate post-chemotherapy fatigue, sleep disturbances, and cognitive dysfunction, underscoring its close link to post-treatment well-being [9].

To alleviate emotional stress in cancer patients, many non-pharmacological treatments have been widely discussed [10]. In recent years, the natural environment has been considered an alternative therapeutic resource for urban people to relax and reduce stress. Recent studies have found that forest bathing or forest therapy has therapeutic effects, such as enhancing health, reducing negative emotions [11], relieving stress, and relaxing the mind [12, 13]. It also helps improve symptoms of depression and anxiety [14, 15], lowers sympathetic nervous system activity, and increases parasympathetic nervous system activity, stabilizing the autonomic nervous system, reducing blood pressure, and improved cognitive function and sleep [16]. For patients with specific mental and physical illnesses, research indicates that forest therapy can alleviate symptoms by reducing depression, decreasing negative emotions [17], and promoting stress recovery [18]. Beyond its psychological effects, other studies have demonstrated that forest bathing also benefits physiological health by enhancing immune [19] function and antioxidant capacity [20], and by increasing alpha and beta brain wave activity [21]. Natural substances released by the forest and phytoncides can improve physical and mental health, reduce depression, and lower cortisol levels, induce deep sleep, and activate natural killer cell function, thereby effectively enhancing immunity [22]. Salivary cortisol levels also tend to decrease following forest therapy [23] or yoga program [24]. Therefore, forest healing improves anxiety and depression evaluations, which can be measured not only through subjective questionnaires but also through cortisol concentration in saliva.

Previous research on natural therapies for cancer patients has primarily relied on subjective questionnaires for assessment [25–30], with few studies collecting objective physiological data [31, 32]. One study on 22 cancer patients found that integrated medical interventions, including forest therapy, improved psychological state and increased NK cell activity [31]. Another study on young cancer patients and caregivers showed that multi-night wilderness trips reduced anxiety, depression, and sleep disturbances while lowering Interleukin-6 (IL-6) levels and slightly increasing C-reactive protein (CRP) [32]. However, these studies did not directly measure emotional stress or isolate forest healing effects, and their complex interventions made detailed analysis challenging.

This study aims to investigate whether guided, programmed forest therapy activities can benefit cancer survivors by alleviating physical and psychological stress. The analysis combines subjective self-reported questionnaires with objectively measured physiological data for a comprehensive evaluation. Through this research, we seek to offer cancer survivors a safe, non-pharmacological complementary therapy that promotes physical and mental relaxation, as well as stress relief. Additionally, the study will provide scientific evidence of the effectiveness of forest healing activities and serve as a reference for future health promotion programs and policy development.

## 2. Materials and methods

### 2.1 Study design

This study employed a prospective single-arm design with a pretest–posttest structure to assess the effects of the intervention. The intervention consisted of a structured two-hour forest therapy program conducted in a submontane forest setting at Fushan Botanical Garden. To evaluate the efficacy of the intervention, both self-reported questionnaires and physiological indicators were collected at two time points: prior to the intervention (baseline, T0) and immediately or subsequently after the intervention (T1).

On the morning of the experiment, participants first completed the questionnaires and then traveled together by car to Fushan Botanical Garden. Before lunch (at 11:30 a.m.), saliva samples were collected, and blood pressure and heart rate were measured; this was designated as the baseline (T0). The two-hour forest therapy session was conducted from 12:30 to 14:30. Immediately after the session, saliva samples were collected again, followed by a 15-minute rest period before measuring blood pressure and heart rate. Participants then completed the *Profile of Mood States, 2nd Edition – Adult Short Form (POMS 2-A)*, which was designated as T1. Finally, all participants returned together by car before 16:00. Due to the differing administration schedules of the questionnaires, the *Beck Anxiety Inventory (BAI)* was completed one week after the intervention, and the *Beck Depression Inventory–Second Edition (BDI-II)* two weeks after, both representing the T1 assessments.

Ethical approval was obtained from the Taipei City Hospital Research Ethics Committee (Approval No. TCHIRB-11104010), with the approval period extending from June 12, 2022, to March 31, 2023. The trial is registered with ClinicalTrials.gov (Identifier: NCT06001723).

### 2.2 Participants

**Fig. 1:**
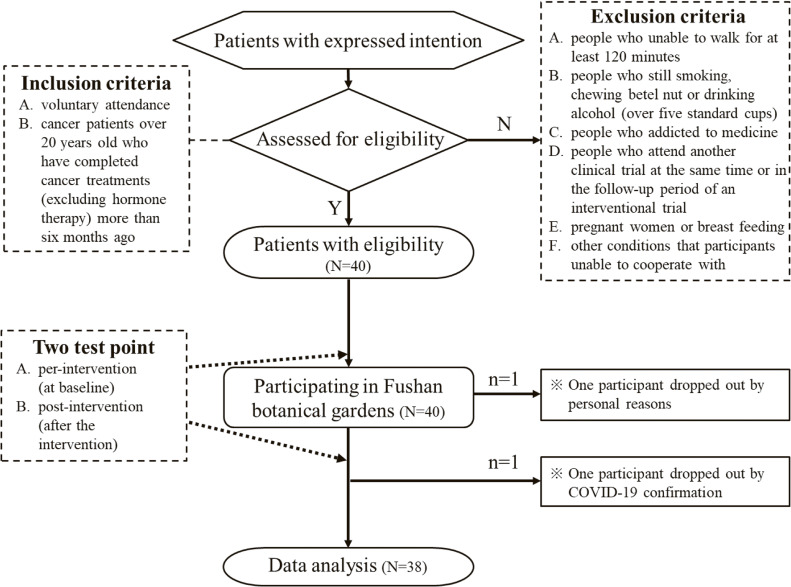
Study flowchart showing design, inclusion and exclusion criteria, and reasons for dropout.

All participants were recruited through posters and online registration forms distributed by the Linsen Chinese Medicine and Kunming Branch (LCMKB) of Taipei City Hospital. An orientation session was held on August 4, 2022, during which the study procedures were explained and informed consent was obtained. Notification letters were subsequently distributed to participants. The forest therapy intervention took place on August 16 and 17, 2022, at the Fushan Botanical Garden, with two cohorts of 20 participants each, for a total of 40 participants (Fig. 1).

Inclusion criteria were as follows: (1) willingness to voluntarily participate in the study; and (2) cancer patients aged 20 years or older who had completed Western medical treatments—such as surgery, radiotherapy, chemotherapy, or targeted therapy (excluding oral hormone therapy)—at least six months prior to enrollment.

Exclusion criteria included: (1) individuals unable to walk continuously for more than 120 minutes, as participants were required to confirm their physical capability to engage in and complete the intervention; (2) current smokers, betel nut chewers, and heavy drinkers (defined as consuming five or more standard drinks on any occasion), due to the high likelihood of comorbid conditions and potential confounding effects; (3) individuals with drug addictions (including both narcotic and non-narcotic substances); (4) individuals currently enrolled in another clinical trial or in the follow-up phase of an interventional study; (5) pregnant or breastfeeding women, due to hormonal differences compared to the general female population; and (6) individuals unable or unwilling to comply with study requirements (e.g., refusal to sign the informed consent form). Participants retained the right to withdraw from the study at any time, and this provision was clearly communicated.

### 2.3 Environmental condition

#### 2.3.1 Fushan Botanical Garden

**Fig. 2:**
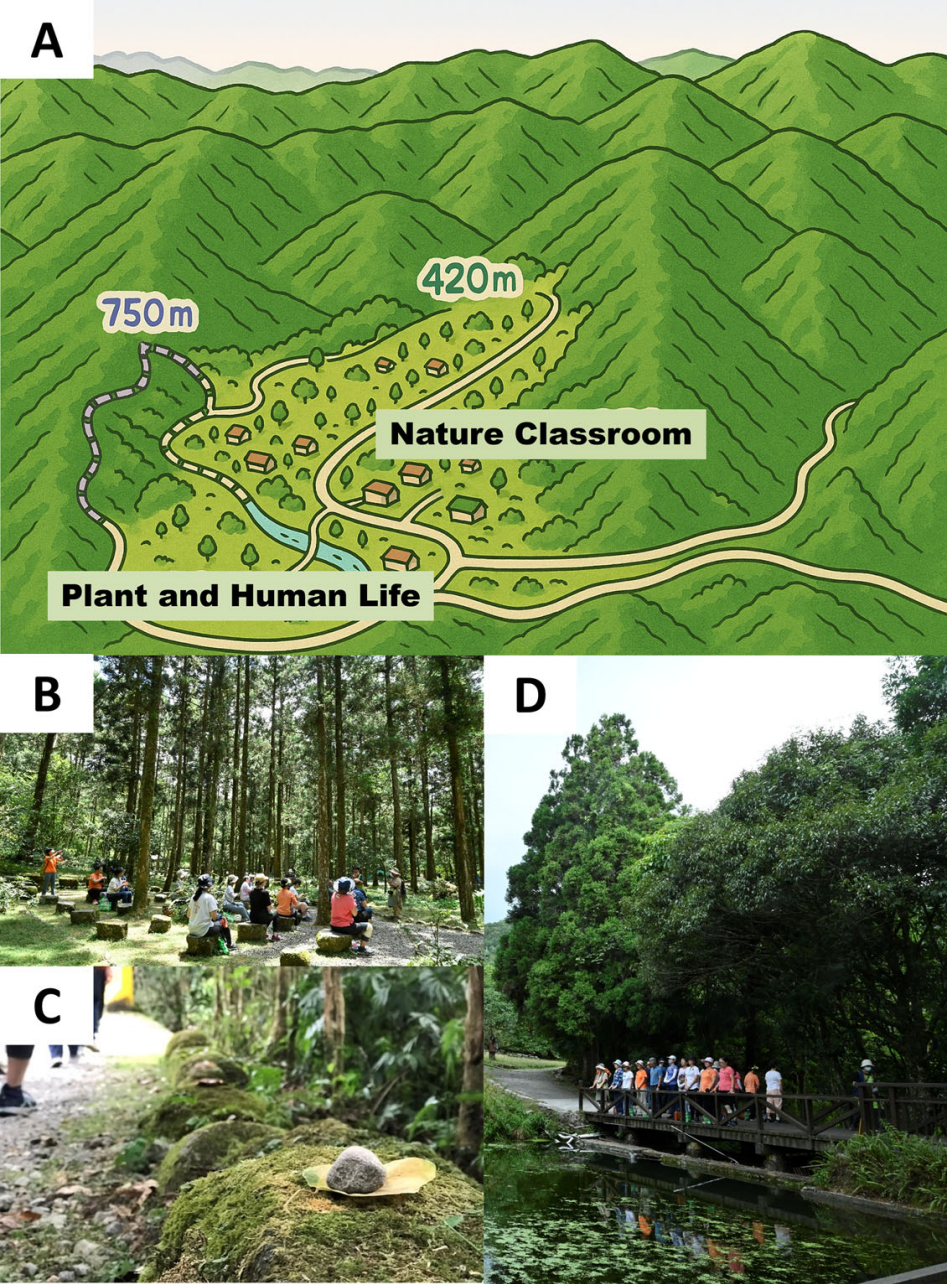
Photo gallery of forest therapy session at Fushan: (A) landscape; (B) gathering; (C) leaf–stone stacking; (D) listening.

The Fushan Botanical Garden is a submontane forest area, located on the border between Wulai District in New Taipei City and Yuanshan Township in Yilan County, Taiwan (24.75767°N, 121.59539°E). Situated in a submontane forest zone, the garden spans elevations ranging from 400 to 1,400 meters above sea level. The area has an average annual temperature of 18.5 °C, an average annual rainfall of 4,125 millimeters, and an average annual relative humidity of 94.1%. The region is characterized by damp and rainy winters with no distinct dry and wet seasons throughout the year. The garden predominantly features natural broadleaf forests, rich in plant and animal species, making it an ideal place for studying natural ecology. The Fushan Experimental Forest has been actively conducting numerous long-term ecological research projects to study the composition and functions of forest ecosystems, including both biotic and abiotic factors. Over a hundred research reports have been published on Fushan, establishing a wealth of fundamental data and providing significant academic and educational value. To balance the natural environment, resource characteristics, and research purposes, the Forestry Research Institute has divided the forest area into the “Water Source Protection Area,” “Botanical Garden Area,” and “Hapen Nature Reserve.” The Botanical Garden covers a total of 409.5 hectares, of which approximately 30 hectares of relatively flat terrain are designated as public plant display areas. The remaining areas are reserved exclusively for research and conservation and are not open to the public. This study will primarily be conducted in the relatively flat “Nature Classroom” and “Plant and Human Life” areas within the Botanical Garden (Fig. 2A).

#### 2.3.2 Atmospheric condition

On August 16, 2022, the weather was partly cloudy to clear, with a temperature of 36.7 °C, humidity at 51.2%, CO_2_ concentration at 471.5 ppm, and noise level at 36.9 dB. Air quality was classified as good, with the following measurements: PM2.5: 14 µg/m^3^, PM10: 12 µg/m^3^, O_3_: 37.9 ppb, CO: 0.18 ppm, NO_2_: 4.4 ppb, and SO_2_: 1.6 ppb. On August 17, 2022, the weather was also partly cloudy to clear, with a temperature of 34.5 °C, humidity at 53.4%, CO_2_ concentration at 567.3 ppm, and noise level at 39.5 dB. Air quality remained good, with the following measurements: PM2.5: 15 µg/m^3^, PM10: 14 µg/m^3^, O_3_: 37.3 ppb, CO: 0.19 ppm, NO_2_: 4.2 ppb, and SO_2_: 1.6 ppb. Temperature, humidity, and noise data were obtained from the botanical garden’s mobile monitoring equipment, while air quality data were sourced from the Taiwan Air Quality Monitoring Network of the Ministry of Environment.

### 2.4 Program of forest therapy

The 2-hour forest therapy activity, led by a horticultural therapist from the Taiwan Horticultural Therapy Association, aims to provide an immersive experience in nature through guided meditation and the cultivation of inner peace [33]. The program includes activities such as leaf grouping, writing one’s plant name, leaf-stone stacking, blindfolded listening, body stretching, fox-walk meditation, blindfolded tree searching, “my tree friend,” earth mandala creation, and experience sharing. A comprehensive description of the activities and their intended purposes is available in Supplementary Material 1. This structured schedule ensures a holistic and engaging forest therapy experience, maximizing its therapeutic benefits and fostering a deep connection with nature for participants. Photos of the forest therapy activities can be found in Fig. 2(B–D).

### 2.5 Measurements

#### 2.5.1 Self-report questionnaire

To evaluate the negative emotions such as anxiety, depression, and emotional state in cancer survivors, we use three kinds of questionnaires to evaluate: the Profile of Mood States 2nd Edition–Adult Short (POMS 2-A) [34], the Beck Depression Inventory-Second edition (BDI-II) [35] and the Beck Anxiety Inventory (BAI) [36].

At baseline (T0) and post-intervention (T1), participants completed paper copies of the POMS 2-A questionnaire, which assesses both negative and positive emotions. The Total Mood Disturbance (TMD) score of the POMS 2-A reflects overall emotional balance, with higher scores indicating a greater proportion of negative emotions and lower scores suggesting fewer negative emotions. The questionnaire consists of 35 items, each describing an emotional state, and participants rate their current feelings. These emotions are categorized into seven domains: Tension-Anxiety (TA), Depression-Dejection (DD), Fatigue-Inertia (FI), Anger-Hostility (AH), Confusion-Bewilderment (CB), Vigour-Activity (VA), and Friendliness (F). The first five categories represent negative emotions, while VA represents positive emotions. F assesses social functioning and is not included in emotional calculations. The TMD score is calculated by summing the scores of the five negative emotion categories (AH + CB + DD + FI + TA) and subtracting the VA score. Each item is rated on a scale from 0 to 4, with the total score ranging from −20 to 100. The scale has demonstrated strong reliability, with Cronbach’s alpha ranging from 0.80 to 0.95 [37].

The Beck Depression Inventory-Second Edition (BDI-II) is a self-report questionnaire designed to assess the severity of depressive symptoms. Originally developed based on patient-reported experiences of depression, it was later adapted for use in non-clinical populations. The BDI-II consists of 21 items, each with four response options ranging from 0 (not at all) to 3 (severe). Participants rate the extent to which they have been affected by each symptom over the past two weeks. The total score reflects the severity of depressive symptoms, categorized as follows: 0–13 (minimal depression), 14–19 (mild depression), 20–28 (moderate depression), and 29–63 (severe depression). The Beck Anxiety Inventory (BAI) is similar to the BDI-II but assesses the severity of anxiety symptoms over the past week. It also consists of 21 items, with a total score ranging from 0 to 63. The Chinese versions of both the BDI-II and BAI were translated and published in Taiwan under authorization from NCS Pearson, Inc. by the Chinese Behavioral Science Corporation. Studies published in Chinese-language journals have validated the reliability and validity of these scales, with Cronbach’s α coefficients of 0.92 for the BDI-II [38] and 0.95 for the BAI [39].

#### 2.5.2 Blood pressure and heart rate variability

At each of the two intervention time points, participants remained seated and rested for 15 minutes before measurement. A wrist-worn physiological monitor (ANSWatch TS-0411) [40, 41] was placed on the upper wrist, and after powering on the device, the measurement was conducted, taking approximately 6–7 minutes to complete. This device measures physiological parameters, including blood pressure, heart rate, and heart rate variability (HRV). It also assesses autonomic nervous system stability through indicators such as normalized unit of high frequencies (nuHF), normalized unit of low frequencies (nuLF), and the low frequency-to-high frequency ratio LF/HF ratio (LF/HF). HF reflects parasympathetic activity, while LF represents sympathetic activity. A decrease in HRV values and an increase in the LF/HF ratio are both associated with impaired cardiovascular function and heightened emotional stress [42, 43].

#### 2.5.3 Salivary cortisol

In this study, psychological and physiological changes, as well as pain-related stress in cancer patients, can influence hormone secretion, with salivary cortisol serving as a key biomarker [23, 24]. Before saliva collection, participants abstained from eating for at least one hour and from alcohol for 12 hours. Samples were collected using unstimulated passive drool into 1.5 mL Eppendorf tubes (≥500 µL) within the recommended diurnal window (11:30–17:30) and stored at −20 °C until analysis. Salivary cortisol was measured using the Salimetrics^®^ Expanded Range High Sensitivity Cortisol ELISA (Catalog No. 1-3002-5; lot 220-8538/230-3540). All samples were assayed in triplicate (CV <20%). Assay performance followed manufacturer specifications, with a detection range of 3.0–0.012 µg/dL and an LLOQ of 0.018 µg/dL.

### 2.6 Statistical analysis

We employed descriptive statistics to describe the baseline characteristics and outcomes of study participants. In the inferential statistics, the changes in outcome measurement between the pre-test and post-test were analyzed using the Wilcoxon signed-rank test, and the effect sizes were estimated with the Wilcoxon R.

To address multiplicity, outcomes were grouped into two families based on types of measurements—psychological distress (POMS total and subscales, BDI-II, BAI), and physiological indicators (heart rate, SBP, DBP, HRV, LF/HF, salivary cortisol). Within each family, we controlled the false discovery rate (FDR) using the Benjamini–Hochberg procedure and report both raw p-values and FDR-adjusted q-values.

We utilized linear regression models to investigate the impact of different demographic factors on the outcome following the forest therapy. The pre-specified demographic variables of interest comprised gender, age, duration of cancer, and baseline value of outcome measurement.

$$Y_{j}=\beta_{0,j}+\sum_{i=1}^{4}\beta_{i,j}X_{i}+\varepsilon_{j}$$

• *j*: POMS-TMD, POMS-AH, POMS-CB, POMS-DD, POMS-FI, POMS-TA, POMS-VA, BDI-II, BAI, and cortisol
• *Y_j_*: changes in outcome measurement
• *X*_1_∼*X*_4_: gender, age, duration of cancer, and grouped by baseline value of outcome measurement
• *β*_0,_*_j_*: intercept
• *β*_1,_*_j_*∼*β*_4,_*_j_*: regression coefficient of each covariate
• ε*_j_*: residual

Each regression coefficient (*β*_1,_*_j_*∼*β*_4,_*_j_*) in the model quantifies the impact on the outcome variable following forest therapy corresponding to a one-unit increase in the respective covariate, adjusting for all other variables in the model. The primary results were visualized using forest plots, depicting the regression coefficients and their 95% confidence intervals. These models were considered exploratory, and no additional multiplicity correction was applied.

All analyses were performed using the available raw data, with no missing-data imputation or additional handling/exclusion of outliers. All participants were included in outcome-specific analyses. Statistical analyses were conducted using SAS (version 9.4; SAS Institute, Cary, NC, USA) and R (version 4.4.3; R Foundation for Statistical Computing, Vienna, Austria). Two-sided significance was set at p < 0.05.

## 3. Result

### 3.1 Characteristics of the participants

Forty cancer survivors were initially enrolled in the forest healing program. After the intervention, one participant withdrew for personal reasons and another due to a confirmed COVID-19 diagnosis, resulting in 38 participants being included in the final analysis. The study procedure is depicted in Fig. 1. The characteristics of the participants in this study are summarized in Table 1. The median age of the participants was 61 years. The median body mass index (BMI) was 22.3 kg/m^2^ (range, 20.7–24.8). Over 80% of the participants had attained tertiary education. Approximately half (44.74%) had a history of breast cancer, and 76.31% were diagnosed at stage I or II. The median duration since cancer diagnosis was 32 months (range, 18.0–54.0), and the median time since the last treatment was 24 months (range, 11.0–45.0), representing a population that had begun to physically recover but remained within the risk of five-year recurrence [6].

**Table 1:** The demographic characteristics of the 38 participants.

**Variable**	**Value^a^ (n = 38)**
**Gender**	
Male	11 (28.95)
Female	27 (71.05)
**Age** (years)	61.0 (52.0, 65.0)
≥65	10 (27.03)
<65	27 (72.97)
**Height** (cm)	161.5 (158.0–165.0)
**Weight** (kg)	59.5 (54.0–66.7)
**BMI** (kg/m^2^)	22.3 (20.7–24.8)
**Education**	
Tertiary education or above^b^	32 (84.21)
Less than tertiary education^c^	6 (15.79)
**Duration of Cancer** (months)	32.0 (18.0–54.0)
**Time from Last therapy** (months)	24.0 (11.0–45.0)
**Type of cancer**	
Breast cancer	17 (44.74)
Other cancer	21 (55.26)
**Stage of cancer**	
I	15 (39.47)
II	14 (36.84)
III	6 (15.79)
IV	3 (7.89)

a. Values are presented as median (interquartile range: Q1–Q3) or number (%).

b. Tertiary education includes college-level education and higher.

c. Less than tertiary education includes high school education and below.

Abbreviations: BMI, body mass index.

### 3.2 Changes in self-reported questionnaires

Table 2 summarizes the outcomes at baseline (T0) and post-intervention (T1), while Table 3 presents the changes in these measures (T1–T0). The effects of the intervention were analyzed using the Wilcoxon signed-rank test, and effect sizes were estimated using the Wilcoxon r.

**Table 2:** The outcomes at two time points: baseline (T0) and post-intervention (T1).

**Outcome**	**Baseline** **(T0)**	**Post-intervention** **(T1)**
POMS 2-A		
Total Mood Disturbance (TMD)	4.0 (1.0, 16.0)	−8.0 (−11.0, −1.0)
Tension-Anxiety (TA)	5.0 (4.0, 8.0)	1.0 (0.0, 3.0)
Depression-Dejection (DD)	1.0 (2.0, 3.0)	1.0 (0.0, 2.0)
Fatigue-Inertia (FI)	5.0 (4.0, 6.0)	1.0 (0.0, 4.0)
Anger-Hostility (AH)	4.5 (3.0, 5.0)	0.5 (0.0, 3.0)
Confusion-Bewilderment (CB)	5.0 (3.0, 5.0)	1.0 (0.0, 3.0)
Vigour-Activity (VA)	14.0 (11.0, 16.0)	14.0 (12.0, 16.0)
Friendliness (F)	16.0 (14.0, 17.0)	18.0 (15.0, 19.0)
BDI-II	5.5 (2.0, 11.0)	4.0 (1.0, 10.0)
BAI	5.0 (2.0, 11.0)	3.5 (0.0, 8.0)
Heart rate (bpm)	75.0 (70.0, 86.0)	79.0 (70.0, 88.0)
SBP (mmHg)	120.0 (110.0, 133.0)	123.0 (113.0, 140.0)
DBP (mmHg)	79.0 (74.0, 92.0)	88.0 (77.0, 94.0)
Heart Rate Variability (ms)	26.0 (18.0, 39.0)	29.5 (24.0, 45.0)
LF/HF	1.17 (0.75, 1.63)	1.15 (0.79, 2.13)
Salivary cortisol (µg/dL)	1.07 (0.79, 1.68)	0.42 (0.22, 1.07)

Data presented as median (interquartile range: Q1, Q3)

Abbreviations: POMS 2-A, Profile of Mood States 2nd Edition–Adult Short; BDI-II, The Beck Depression Inventory-Second edition; BAI, The Beck Anxiety Inventory; SBP, systolic blood pressure; DBP, diastolic blood pressure; LF/HF, low frequency-to-high frequency ratio.

**Table 3:** The changes in outcome measures of the intervention (T1 to T0).

**Outcome**	**Effectiveness of the intervention** **(T1 to T0)**	**p-value^a^**	**q-value^b^**		**Effect size** **(Wilconxon r)**	**magnitude^c^**
POMS 2-A						
Total Mood Disturbance (TMD)	−14.0 (−19.0, −6.0)	<0.0001***	0.0002***	psychological distress^d^	0.778	large
Tension-Anxiety (TA)	−4.0 (−6.0, −1.0)	<0.0001***	0.0002***		0.794	large
Depression-Dejection (DD)	−1.0 (−2.0, 0.0)	0.0005***	0.0008***		0.578	large
Fatigue-Inertia (FI)	−3.0 (−5.0, −1.0)	<0.0001***	0.0002***		0.813	large
Anger-Hostility (AH)	−3.0 (−4.0, 0.0)	<0.0001***	0.0002***		0.750	large
Confusion-Bewilderment (CB)	−2.0 (−4.0, −1.0)	<0.0001***	0.0002***		0.719	large
Vigour-Activity (VA)	0.0 (−1.0, 2.0)	0.2655	0.2655		0.154	small
Friendliness (F)	1.0 (0.0, 3.0)	0.0006***	0.0009***		0.501	large
BDI-II	−1.0 (−2.0, 0.0)	0.0013***	0.0014**		0.519	large
BAI	−2.0 (−5.0, 0.0)	0.001***	0.0013**		0.495	moderate

Heart rate (bpm)	0.0 (−3.0, 6.0)	0.6879	0.7815	physiological indicators^e^	0.062	small
SBP (mmHg)	4.0 (−3.0, 11.0)	0.0159*	0.0477*		0.384	moderate
DBP (mmHg)	2.5 (−2.0, 11.0)	0.0554	0.1108		0.316	moderate
Heart Rate Variability (ms)	2.0 (−5.0, 9.0)	0.3861	0.5792		0.146	small
LF/HF	−0.03 (−0.77, 1.06)	0.7815	0.7815		0.046	small
Salivary cortisol (µg/dL)	−0.61 (−1.08, −0.24)	<0.0001***	0.0006***		0.872	large

Data presented as median (interquartile range: Q1, Q3)

Note. a: Wilcoxon signed-rank test, b: q-values represent p-values corrected for FDR using the Benjamini–Hochberg procedure, c: small with 0 to 0.3, moderate with 0.3 to 0.5, large with 0.5 to 1, *p(q) <0.05 with statistical significance, **p(q) <0.01 with statistical significance, ***p(q) <0.001 with statistical significance, d: psychological distress (POMS total and subscales, BDI-II, BAI), e: physiological indicators (heart rate, SBP, DBP, HRV, LF/HF, salivary cortisol)

Abbreviations: POMS 2-A, Profile of Mood States 2nd edition–Adult Short; BDI-II, The Beck Depression Inventory-Second edition; BAI, The Beck Anxiety Inventory; SBP, systolic blood pressure; DBP, diastolic blood pressure; LF/HF, low frequency-to-high frequency ratio LF/HF ratio.

Cancer survivors demonstrated significant reductions (p < 0.001) in anxiety, depression, and mood disturbance scores, as measured by the BAI, BDI-II, and POMS 2-A questionnaires. The median TMD score on the POMS 2-A decreased from 4.0 (IQR: 1.0, 16.0) at baseline to −8.0 (IQR: −11.0, −1.0) post-intervention, accompanied by significant decreases with large effect sizes in the subscales of Tension–Anxiety, Fatigue–Inertia, Anger–Hostility, and Confusion–Bewilderment. Scores for Vigour–Activity remained stable, whereas Friendliness scores increased from 16.0 (IQR: 14.0, 17.0) to 18.0 (IQR: 15.0, 19.0), also with a large effect size. Similar trends were observed for both the BDI-II and BAI. Median BDI-II scores decreased from 5.5 (IQR: 2.0–11.0) to 4.0 (IQR: 1.0–10.0), remaining within the “minimal” severity category. Median BAI scores similarly decreased from 5.0 (IQR: 2.0–11.0) to 3.5 (IQR: 0.0–8.0), also within the “minimal” range. Although both measures stayed in the minimal severity category, the magnitude of change corresponded to large effect sizes based on the Wilcoxon r.

### 3.3 Changes in objective physiological indicators

Based on data from Tables 2 and 3, physiological indicators showed a slight post-intervention increase in heart rate and blood pressure, with median SBP rising from 120.0 mmHg to 123.0 mmHg and DBP from 79.0 mmHg to 88.0 mmHg. Heart rate variability parameters also improved, with the median value increasing from 26.0 ms to 29.5 ms, while the median LF/HF ratio slightly decreased from 1.17 to 1.15. Among these measures, only SBP demonstrated a statistically significant change (q < 0.05) with a moderate effect size. In contrast, salivary cortisol concentrations declined substantially from 1.07 µg/dL (IQR: 0.79, 1.68) to 0.42 µg/dL (IQR: 0.22, 1.07), reflecting a highly significant reduction (q < 0.001) with a large effect size, indicating a substantial decrease in physiological stress following the intervention.

### 3.4 Linear regression and forest plots

**Fig. 3:**
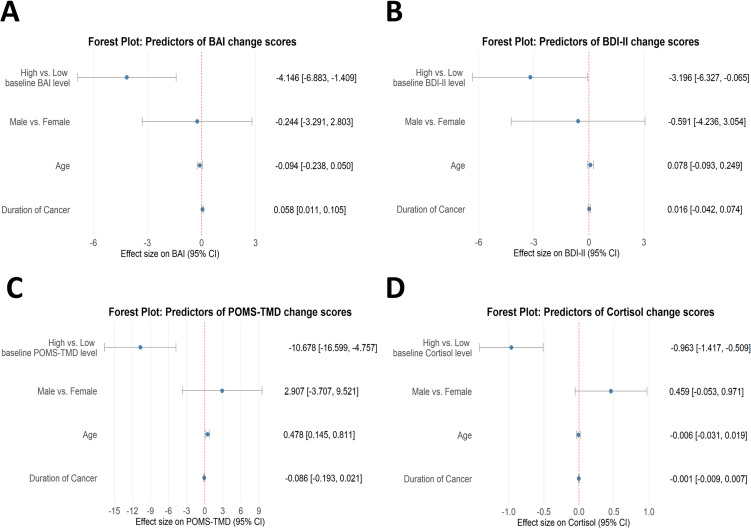
Forest plots of demographic and baseline factors influencing pre–post changes: (A) BAI; (B) BDI-II; (C) POMS-TMD; (D) salivary cortisol.

Figure 3 and Supplementary Material 2 present forest plots and linear regression analyses that examine the influence of demographic characteristics and baseline outcome measures on the magnitude of change observed between pre- and post-intervention assessments. The overall results suggest that forest therapy may confer greater benefits to individuals with higher initial levels of psychological distress or elevated physiological stress markers. Moreover, the analyses indicate a possible trend whereby participants with shorter cancer duration, younger age, or male gender tend to experience relatively larger improvements following the intervention, as reflected in certain scale outcomes.

For psychological distress, both BAI and BDI-II demonstrated significant associations with participant characteristics. BAI change scores were positively correlated with cancer duration (β = 0.058, p = 0.017), indicating that each additional month of cancer corresponded to a 0.058-point increase in BAI change, reflecting a reduced intervention effect over time. Conversely, participants with baseline BAI scores above the median experienced significantly greater anxiety reductions (β = −4.146, p = 0.004), implying that forest therapy conferred enhanced benefits for those with more severe initial anxiety. A similar pattern was observed for BDI-II scores, where participants with baseline values above the median showed significantly larger decreases (β = −3.196, p = 0.044), indicating stronger therapeutic effects among individuals with higher baseline depression. Regarding emotional states, changes in POMS-TMD were significantly associated with age (β = 0.478, p = 0.006), whereby each additional year corresponded to a 0.478-point increase in POMS-TMD change. Participants with baseline POMS-TMD scores above the median experienced markedly greater mood improvements (β = −10.678, p = 0.001), indicating more pronounced effects in younger individuals and those with higher baseline mood disturbance.

In terms of physiological indicators, responses were similarly influenced by baseline values. Participants with baseline cortisol levels above the median exhibited significantly greater reductions post-intervention (β = −0.963, p = 0.0001), highlighting the effectiveness of forest therapy in individuals with elevated baseline cortisol. This finding corroborates the psychological results, suggesting that the intervention may be particularly beneficial for those with higher initial stress levels.

## 4. Discussion

### 4.1 Main finding of the study

Significant reductions were observed in total mood disturbance (TMD) scores and several subscales of the POMS 2-A, including POMS-TA, POMS-DD, POMS-AH, POMS-CB, and POMS-FI, indicating an overall alleviation of negative emotional states. Notably, scores for POMS-VA remained stable post-intervention, suggesting that the forest therapy activities did not contribute to participant fatigue. Consistent with these findings, psychological distress—as measured by BAI and BDI-II—declined significantly, indicating reductions in anxiety and depressive symptoms following the intervention.

In addition to significant improvements in self-reported questionnaires, certain physiological stress biomarkers also exhibited favorable changes. Specifically, salivary cortisol levels showed a significant decrease after the intervention (median difference = −1.54, p < 0.0001). However, measurements obtained from the wrist-worn physiological monitor revealed no significant changes in heart rate (HR), diastolic blood pressure (DBP), heart rate variability (HRV), or the low frequency-to-high frequency ratio (LF/HF). A slight increase in systolic blood pressure (SBP) was detected, potentially due to an insufficient resting period before measurement or elevated emotional arousal during the post-activity sharing session. Interestingly, a modest, though not statistically significant, increase in HRV accompanied by a decrease in the LF/HF ratio was observed. This pattern, which contrasts with changes typically associated with cardiovascular disease or stress responses [42, 43], may suggest potential benefits of forest therapy.

### 4.2 The role of baseline and demographic factors in influencing intervention outcomes

Linear regression analysis revealed significant associations between emotional and physiological variables in cancer survivors and underscored the predictive value of baseline conditions, age, gender, and other factors for treatment outcomes. These findings provide preliminary insights for the development of more personalized intervention strategies. The regression results were further illustrated using forest plots, with key observations as follows:

**Baseline Distress Levels:** Participants with higher baseline psychological distress, as measured by POMS 2-A, BAI, and BDI-II, or elevated physiological stress markers such as salivary cortisol, tended to show greater improvements following the intervention. Although causality cannot be established, these findings underscore the potential value of assessing pre-intervention symptom levels to identify individuals who may benefit most. In future forest therapy programs, this approach could inform participant selection—for example, focusing on breast cancer patients with a higher prevalence of depressive symptoms [7, 8] or using brief screening questionnaires to identify cancer patients with elevated stress or negative affect.

**Age and Cancer Duration:** Younger participants tended to show greater improvements in emotional outcomes, as measured by POMS-TMD, which may reflect higher psychological adaptability or greater receptivity to nature-based interventions. Although further research is needed, adapting the content or delivery of interventions for older adults could help enhance accessibility and effectiveness across age groups. Shorter cancer duration was modestly associated with better emotional responses, as measured by BAI, possibly reflecting lower emotional fatigue or greater openness to new interventions. While not a primary predictor, cancer duration remains an important contextual factor to consider in intervention planning. In line with previous research indicating that the first year after a cancer diagnosis carries the greatest risk of depression [8], earlier implementation of forest therapy—assuming patients are physically able to participate—may yield more favorable emotional outcomes.

Overall, these exploratory findings suggest that individual characteristics—particularly baseline distress levels—may influence the extent of benefit derived from forest therapy. Integrating these factors into the design and delivery of interventions could enhance their relevance and efficacy. As this study offers initial evidence within the Taiwanese context, further research is warranted to validate these associations and refine stratified approaches to nature-based care for cancer survivors.

### 4.3 Limitations

#### Absence of a control group:

This study employed a single-arm pre–post design without a concurrent control group. Therefore, the observed changes cannot be attributed exclusively to the forest therapy intervention. In the absence of a comparator, the potential influence of natural recovery, expectancy or Hawthorne effects, regression to the mean, and other psychosocial factors cannot be fully excluded. We sincerely acknowledge this as an important limitation and recommend that future research adopt randomized controlled or waitlist-controlled designs to enhance internal validity and better isolate the true intervention effects.

#### Time-of-day confounding for cortisol:

Salivary cortisol follows a well-established diurnal rhythm, typically exhibiting a gradual decline throughout the day. In this study, sampling was conducted within controlled time windows to reduce circadian-related variability: baseline (T0) samples were collected before lunch at 11:30 a.m., and post-intervention (T1) samples were collected around 14:30 p.m. Nevertheless, in the absence of a control or comparison group, it remains possible that part of the observed reduction in cortisol levels reflects this natural diurnal slope rather than the forest therapy itself. A review of the relevant literature suggests that diurnal changes in cortisol (morning-to-afternoon differences) are influenced not only by stress but also by individual factors such as BMI and education level [44], making it challenging to apply a simple time-based correction. Daniel (2012) reported that education and Δ cortisol were inversely related to BMI, and that higher education buffered the magnitude of the association between Δ cortisol and BMI. In the present study, 71% of participants were female and 84% had attained tertiary or higher education, which may suggest that the influence of diurnal cortisol variation on our findings was relatively limited.

#### Sample size and generalizability:

The relatively small sample size (n = 38) limits statistical power and generalizability. In addition, convenience-based recruitment resulted in a sample with a high proportion of female participants (71%) and individuals with tertiary education or above (84%). This demographic composition may not be representative of the broader cancer survivor population and introduces potential selection bias. Consequently, the applicability of our findings to populations with different gender distributions, educational backgrounds, or socioeconomic characteristics is limited. Future studies using randomized or stratified sampling strategies, conducted across multiple sites with more diverse participant groups, are needed to strengthen external validity and further verify the effects of forest therapy in cancer survivors.

#### Potential impact of external events:

Data collection took place during the COVID-19 pandemic, a period of heightened uncertainty and psychological stress. Such contextual factors may have influenced participants’ emotional states independently of the intervention, particularly regarding concerns about cancer recurrence. These influences could confound interpretations of psychological outcomes, including the BDI-II.

### 4.4 Rationale

This study assessed the effectiveness of a structured, professionally guided forest therapy program for cancer survivors by combining self-reported outcomes with physiological indicators. The observed reductions in negative emotions and stress markers highlight its therapeutic potential and address a literature gap where psychological and physiological effects are often examined separately.

While most Asian research has been conducted in Japan and South Korea [11, 14], no prior studies have targeted cancer survivors in Taiwan. With 2.197 million hectares of forest and a coverage rate of 60.71%—nearly double the global average—Taiwan offers an ideal setting. This work complements findings from neighboring countries and provides the first empirical evidence in the Taiwanese context.

### 4.5 Prospections

In 2007, the American College of Sports Medicine (ACSM) and the American Medical Association (AMA) jointly launched the *Exercise is Medicine* (EIM) initiative to encourage healthcare providers to integrate physical activity into routine counseling and chronic disease management. Since then, exercise prescriptions have gained steady clinical adoption, driven by growing evidence and supportive policies.

In 2015, the U.S. National Park Service, in collaboration with medical institutions and community health centers, established *National ParkRx Day*, advancing the “park prescription” movement to promote nature-based physical activity. In Canada, the BC Parks Foundation introduced the nation’s first formal park prescription program in 2020, enabling physicians to prescribe “nature time”—typically 20 minutes per session, totaling at least two hours weekly.

These initiatives reflect a broader shift toward embedding physical activity and nature exposure in preventive healthcare, especially for chronic disease management and mental health promotion. Evidence shows that park prescription interventions can enhance psychological quality of life in healthy middle-aged adults [45], while exercise prescriptions can help alleviate depression and anxiety in lung cancer patients [46]. Given the mounting evidence for the psychological and physiological benefits of forest therapy, implementing forest-based or “green” prescriptions offers a promising, non-pharmacological strategy to improve public health and well-being.

## 5. Conclusion

This study provides preliminary evidence that guided forest therapy may serve as an effective complementary approach to improving psychological well-being and reducing physiological stress among cancer survivors. Significant improvements were observed in both self-reported emotional distress (POMS 2-A, BAI, BDI-II) and objective stress indicators (salivary cortisol). These effects were influenced by baseline psychological and physiological status, age, and cancer duration, with greater benefits seen in participants who had higher initial anxiety or depression, were younger, or had shorter cancer duration. These findings highlight the value of considering baseline health profiles and demographic characteristics when designing forest therapy interventions. Future controlled studies with larger and more diverse samples are needed to confirm these results and support clinical integration in cancer survivorship care.

## Abbreviations

ACSM: American College of Sports Medicine
AH: Anger-Hostility
AMA: American Medical Association
ASCO: American Society of Clinical Oncology
BAI: The Beck Anxiety Inventory
BDI-II: The Beck Depression Inventory-Second edition
BMI: Body Mass Index
CB: Confusion-Bewilderment
COVID-19: Coronavirus disease 2019
CRP: C-reactive protein
DBP: Diastolic Blood Pressure
DD: Depression-Dejection
EIM: Exercise Is Medicine
F: Friendliness
FDR: False Discovery Rate
FI: Fatigue-Inertia
HRV: Heart Rate Variability
HF: Normalized Unit of High Frequencies
IARC: International Agency for Research on Cancer
IL-6: Interleukin-6
LCMKB: Linsen Chinese Medicine and Kunming Branch
LF: Normalized Unit of Low Frequencies
LF/HF: Low Frequency-to-High Frequency Ratio
POMS 2-A: Profile of Mood States 2nd edition–Adult Short
SBP: Systolic Blood Pressure
TA: Tension-Anxiety
TMD: Total Mood Disturbance
VA: Vigour-Activity
WHO: World Health Organization

## Supplementary information

**Additional file 1:** **Supplementary Material 1.** Detailed descriptions of the forest therapy activities and their corresponding therapeutic objectives.

## Declarations

### Availability of data and materials

The datasets generated and/or analyzed during the current study are available from the corresponding author on reasonable request, subject to ethical and privacy considerations.

## References

[1] World Health Organization. Global cancer burden growing, amidst mounting need for services. 2024. Available from: https://www.who.int/news/item/01-02-2024-global-cancer-burden-growing--amidst-mounting-need-for-services. PMC11115397 38438207

[2] Taiwan Ministry of Health and Welfare. Analysis of the Causes of Death Statistics for the Population in 2022. 2022. Available from: https://www.mohw.gov.tw/cp-16-74869-1.html.

[3] Pituskin E. Cancer as a new chronic disease: Oncology nursing in the 21st Century. Can Oncol Nurs J. 2022;32(1):87–92. 35280062 PMC8849169

[4] Ehlers SL, Davis K, Bluethmann SM, . Screening for psychosocial distress among patients with cancer: implications for clinical practice, healthcare policy, and dissemination to enhance cancer survivorship. Transl Behav Med. 2019;9(2):282–91. doi: 10.1093/tbm/iby123. 30566662 PMC6610173

[5] Andersen BL, Lacchetti C, Ashing K, Berek JS, Berman BS, Bolte S, . Management of Anxiety and Depression in Adult Survivors of Cancer: ASCO Guideline Update. J Clin Oncol. 2023 Jun 20;41(18):3426–53. doi: 10.1200/JCO.23.00293. 37075262

[6] Morgan E, O’Neill C, Bardot A, . Collecting Long-Term Outcomes in Population-Based Cancer Registry Data: The Case of Breast Cancer Recurrence. JCO Glob Oncol. 2024;10:e2400249. doi: 10.1200/GO-24-00249. 39481072

[7] Pilevarzadeh M, Amirshahi M, Afsargharehbagh R, Rafiemanesh H, Hashemi SM, Balouchi A. Global prevalence of depression among breast cancer patients: a systematic review and meta-analysis. Breast Cancer Res Treat. 2019;176(3):519–33. doi: 10.1007/s10549-019-05271-3. 31087199

[8] Choi HL, Jeong SM, Jeon KH, . Depression risk among breast cancer survivors: a nationwide cohort study in South Korea. Breast Cancer Res. 2024 Nov 7;26(1):188. doi: 10.1186/s13058-024-01948-w. 39731197 PMC11674164

[9] Jakovljevic K, Kober KM, Block A, Cooper BA, Paul SM, Hammer MJ, . Higher Levels of Stress Are Associated With a Significant Symptom Burden in Oncology Outpatients Receiving Chemotherapy. J Pain Symptom Manage. 2021 Jan;61(1):24–31.e4. doi: 10.1016/j.jpainsymman.2020.07.019. 32721501 PMC7770050

[10] Cifu G, Power MC, Shomstein S, Arem H. Mindfulness-based interventions and cognitive function among breast cancer survivors: a systematic review. BMC Cancer. 2018;18(1):1163. doi: 10.1186/s12885-018-5065-3. 30477450 PMC6260900

[11] Hansen MM, Jones R, Tocchini K. Shinrin-Yoku (Forest Bathing) and Nature Therapy: A State-of-the-Art Review. Int J Environ Res Public Health. 2017;14(8):851. doi: 10.3390/ijerph14080851. 28788101 PMC5580555

[12] Antonelli M, Barbieri G, Donelli D. Effects of forest bathing (shinrin-yoku) on levels of cortisol as a stress biomarker: a systematic review and meta-analysis. Int J Biometeorol. 2019;63(8):1117–34. doi: 10.1007/s00484-019-01717-x. 31001682

[13] Saito H, Horiuchi M, Takayama N, Fujiwara A. Effects of managed forest versus unmanaged forest on physiological restoration from a stress stimulus, and the relationship with individual traits. J For Res. 2019;24(2):77–85. doi: 10.1080/13416979.2019.1586300.

[14] Lee I, Choi H, Bang KS, Kim S, Song M, Lee B. Effects of Forest Therapy on Depressive Symptoms among Adults: A Systematic Review. Int J Environ Res Public Health. 2017;14(3):321. doi: 10.3390/ijerph14030321. 28335541 PMC5369157

[15] Chun MH, Chang MC, Lee SJ. The effects of forest therapy on depression and anxiety in patients with chronic stroke. Int J Neurosci. 2017;127(3):199–203. doi: 10.3109/00207454.2016.1170015. 27033879

[16] Jimenez MP, DeVille NV, Elliott EG, Schiff JE, Wilt GE, Hart JE, James P. Associations between Nature Exposure and Health: A Review of the Evidence. Int J Environ Res Public Health. 2021 Apr 30;18(9):4790. doi: 10.3390/ijerph18094790. 33946197 PMC8125471

[17] Furuyashiki A, Tabuchi K, Norikoshi K, Kobayashi T, Oriyama S. A comparative study of the physiological and psychological effects of forest bathing (Shinrin-yoku) on working age people with and without depressive tendencies. Environ Health Prev Med. 2019;24(1):46. doi: 10.1186/s12199-019-0800-1. 31228960 PMC6589172

[18] Dolling A, Nilsson H, Lundell Y. Stress recovery in forest or handicraft environments – An intervention study. Urban For Urban Green. 2017;27:162–72. doi: 10.1016/j.ufug.2017.07.006.

[19] Tsao TM, Tsai MJ, Hwang JS, Cheng WF, Wu CF, Chou CK, Su TC. Health effects of a forest environment on natural killer cells in humans: an observational pilot study. Oncotarget. 2018 Mar 27;9(23):16501–11. doi: 10.18632/oncotarget.24741. 29662662 PMC5893257

[20] Chun MH, Chang MC, Lee SJ. The effects of forest therapy on depression and anxiety in patients with chronic stroke. Int J Neurosci. 2017 Mar;127(3):199–203. doi: 10.3109/00207454.2016.1170015. 27033879

[21] Hassan A, Tao J, Li G, Jiang M, Aii L, Zhihui J, Zongfang L, Qibing C. Effects of Walking in Bamboo Forest and City Environments on Brainwave Activity in Young Adults. Evid Based Complement Alternat Med. 2018 Feb 11;2018:9653857. doi: 10.1155/2018/9653857. 29785198 PMC5896408

[22] Lee MM, Park BJ. Effects of Forest Healing Program on Depression, Stress and Cortisol Changes of Cancer Patients. J People Plants Environ. 2020;23(2):245–54.

[23] Qiu Q, Yang L, He M, . The Effects of Forest Therapy on the Blood Pressure and Salivary Cortisol Levels of Urban Residents: A Meta-Analysis. Int J Environ Res Public Health. 2022;20(1):458. doi: 10.3390/ijerph20010458. 36612777 PMC9819785

[24] Vadiraja HS, Raghavendra RM, Nagarathna R, Nagendra HR, Rekha M, Vanitha N, . Effects of a yoga program on cortisol rhythm and mood states in early breast cancer patients undergoing adjuvant radiotherapy: a randomized controlled trial. Integr Cancer Ther. 2009 Mar;8(1):37–46. doi: 10.1177/1534735409331456. Erratum in: Integr Cancer Ther. 2009 Jun;8(2):195. 19190034

[25] Scates D, Dickinson JI, Sullivan K, Cline H, Balaraman R. Using Nature-Inspired Virtual Reality as a Distraction to Reduce Stress and Pain Among Cancer Patients. Environ Behav. 2020;52:895–918.

[26] Pearson AL, Breeze V, Reuben A, Wyatt G. Increased Use of Porch or Backyard Nature during COVID-19 Associated with Lower Stress and Better Symptom Experience among Breast Cancer Patients. Int J Environ Res Public Health. 2021;18(17):9102. doi: 10.3390/ijerph18179102. 34501691 PMC8430585

[27] Morris SL, Newhouse I, Larocque T, Gillis KJ, Smith L, Nisbet EK. Becoming One with Nature: A Nature Intervention for Individuals Living with Cancer Participating in a Ten-Week Group Exercise and Wellness Program. Int J Exerc Sci. 2021;14(3):498–518. doi: 10.70252/SOTE9757. 34055162 PMC8136560

[28] Albers T, Weiss LA, Sleeman SHE, Husson O. Evaluation of a Positive Psychology Group Intervention in Nature for Young Cancer Survivors to Promote Well-Being and Post-Cancer Identity Development. J Adolesc Young Adult Oncol. 2021;10(6):726–34. doi: 10.1089/jayao.2020.0147. 33601973

[29] Park KH, Lee H, Park EY, . Effects of an urban forest healing program on cancer-related fatigue in cancer survivors. Support Care Cancer. 2023;32(1):4. doi: 10.1007/s00520-023-08214-3. 38051396

[30] Kim MK, Park HJ, Lee KJ. Living lab modelling as a pilot study assessing the potential psychological health benefits of forest environment for cancer survivors. Obstet Gynecol Sci. 2024;67(4):404–13. doi: 10.5468/ogs.24035. 38987994 PMC11266852

[31] Nakau M, Imanishi J, Imanishi J, . Spiritual care of cancer patients by integrated medicine in urban green space: a pilot study. Explore (NY). 2013;9(2):87–90. doi: 10.1016/j.explore.2012.12.002. 23452710

[32] Victorson D, Doninger G, Victorson S, . Psychosocial and Biological Outcomes of Immersive, Mindfulness-Based Treks in Nature for Groups of Young Adults and Caregivers Affected by Cancer: Results from a Single Arm Program Evaluation from 2016–2021. Int J Environ Res Public Health. 2021;18(23):12622. doi: 10.3390/ijerph182312622. 34886348 PMC8657001

[33] Fan SW, Liu YC, Li JW, Wei RS, Chu YS. [Botanical garden healing during the pandemic]. Forestry Research Newsletter. 2022;29(5):32–8 [Chinese Only]. https://www.airitilibrary.com/Article/Detail?DocID=16056922-202210-202212210015-202212210015-32-38.

[34] Heuchert JP, McNair DM. (No year specified.). Profile of Mood States 2nd Edition™ (POMS) [Database record]. *APA PsycTests*. Available from: doi: 10.1037/t05057-000.

[35] Beck AT, Steer RA, Brown G. Beck Depression Inventory–II (BDI-II) [Database record]. APA PsycTests. 1996. doi: 10.1037/t00742-000.

[36] Beck AT, Epstein N, Brown G, Steer RA. An inventory for measuring clinical anxiety: psychometric properties. J Consult Clin Psychol. 1988 Dec;56(6):893–7. doi: 10.1037//0022-006x.56.6.893. 3204199

[37] Lin S, Hsiao YY, Wang M. Test Review: The Profile of Mood States 2nd Edition. J Psychoeduc Assess. 2014;32(3):273–7. doi: 10.1177/0734282913505995.

[38] Pan AW, Hsu WL. [Application of Rasch Measurement Model in the Construct Validity of the Beck Depression Inventory-II]. Formosan J Med. 2008;12(3):284–91 [Chinese Only]. doi: 10.6320/FJM.2008.12(3).03.

[39] Che HH, Lu ML, Chen HC, Chang SW, Lee YJ. [Validation of the Chinese Version of the Beck Anxiety Inventory]. Formosan J Med. 2006;10(4):447–54 [Chinese Only]. doi: 10.6320/FJM.2006.10(4).05.

[40] Liao CK, Tsai JS, Lin LY, Lee SC, Lai CF, Ho TW, Lai F. Characteristics of Harmonic Indexes of the Arterial Blood Pressure Waveform in Type 2 Diabetes Mellitus. Front Bioeng Biotechnol. 2020 Jul 8;8:638. doi: 10.3389/fbioe.2020.00638. 32733859 PMC7360801

[41] Liao YH, Tai CJ, Ming JL, Lin LH, Chien LY. The Effectiveness of Low-Level LED Light Therapy for Sleep Problems, Psychological Symptoms, and Heart Rate Variability in Shift-Work Nurses: A Randomized Controlled Trial. J Nurs Manag. 2025 Jun 17;2025;6478834. doi: 10.1155/jonm/6478834. 40557249 PMC12187440

[42] Vrijkotte TG, van Doornen LJ, de Geus EJ. Effects of work stress on ambulatory blood pressure, heart rate, and heart rate variability. Hypertension. 2000;35(4):880–6. doi: 10.1161/01.hyp.35.4.880. 10775555

[43] Fang SC, Wu YL, Tsai PS. Heart Rate Variability and Risk of All-Cause Death and Cardiovascular Events in Patients With Cardiovascular Disease: A Meta-Analysis of Cohort Studies. Biol Res Nurs. 2020;22(1):45–56. doi: 10.1177/1099800419877442. 31558032

[44] Daniel M, Moore DS, Decker S, Belton L, DeVellis B, Doolen A, Campbell MK. Associations among education, cortisol rhythm, and BMI in blue-collar women. Obesity (Silver Spring). 2006 Feb;14(2):327–35. doi: 10.1038/oby.2006.42. 16571860

[45] Müller-Riemenschneider F, Petrunoff N, Yao J, . Effectiveness of prescribing physical activity in parks to improve health and wellbeing - the park prescription randomized controlled trial. Int J Behav Nutr Phys Act. 2020;17(1):42. doi: 10.1186/s12966-020-00941-8. 32183815 PMC7079356

[46] Lei J, Yang J, Dong L, . An exercise prescription for patients with lung cancer improves the quality of life, depression, and anxiety. Front Public Health. 2022;10:1050471. doi: 10.3389/fpubh.2022.1050471. 36466452 PMC9714027

